# Estratégia Farmacoinvasiva no Infarto do Miocárdio com Supradesnível do Segmento ST no Brasil: Sexo Feminino como Fator Prognóstico

**DOI:** 10.36660/abc.20220688

**Published:** 2022-11-01

**Authors:** Fernando Cesena

**Affiliations:** 1 Cenocor Guarulhos SP Brasil Cenocor, Guarulhos, SP – Brasil

**Keywords:** Infarto do Miocárdio com Supradesnível do Segmento ST, Terapia Trombolítica, Intervenção Coronária Percutânea, Diferenças Sexuais

A intervenção coronária percutânea primária (ICP) é a terapia recomendada em pacientes com infarto agudo do miocárdio com supradesnível do segmento ST (IAMCSST). Em locais sem capacidade imediata de ICP ou na presença de uma demora antecipada da apresentação hospitalar à ICP primária > 120 minutos, a fibrinólise é indicada. Nesses casos, a angiografia invasiva e a ICP 3 a 24 horas após a fibrinólise podem melhorar o prognóstico e são recomendadas como classe 2a pela recente Diretriz para Revascularização da Artéria Coronária do ACC/AHA/SCAI.^1^ A justificativa para ICP de rotina precoce após fibrinólise, a chamada estratégia farmacoinvasiva, é que a terapia lítica fornece fluxo sanguíneo adequado (grau TIMI 3) em apenas 50-60% dos casos. A ICP pode então aliviar a estenose residual e restaurar o fluxo normal, o que está relacionado ao benefício da reperfusão em reduzir mortalidade. É importante ressaltar que a angiografia invasiva precoce deve seguir a fibrinólise independentemente da resolução do supradesnível do segmento ST, uma vez que as alterações do eletrocardiograma têm baixa precisão na identificação de reperfusão adequada.^2^

A recomendação para a estratégia farmacoinvasiva é apoiada por vários ensaios clínicos controlados e randomizados (ECRs) e meta-análises que demonstram benefícios clínicos em relação à terapia padrão anterior.^3^ Além disso, o estudo STREAM forneceu evidências de que a fibrinólise pré-hospitalar seguida de ICP após 6 a 24 horas é tão eficaz quanto a ICP primária em pacientes com IAMCSST que não podem ser submetidos à ICP primária dentro de 1 hora após o diagnóstico.^4,5^

Os Arquivos Brasileiros de Cardiologia publicam um artigo relatando minuciosamente a experiência de um hospital universitário brasileiro com a abordagem farmacoinvasiva no IAMCSST.^6^ Embora este trabalho observacional seja completamente diferente dos ECRs pregressos em relação ao desenho, propósito e população do estudo, é tentador fazer algumas comparações.

A mortalidade intra-hospitalar no estudo brasileiro foi de 5,6%, superior à mortalidade em 30 dias de 3,3% relatada em metanálise de ECRs^3^ mas não tão superior à mortalidade de 4,6% em 30 dias no braço farmacoinvasivo do estudo STREAM.^4^ Digno de nota, o tempo desde o início dos sintomas até o tratamento fibrinolítico foi maior no estudo brasileiro (mediana de 222 minutos) do que no estudo STREAM (mediana de 100 minutos)^4^ ou nos ECRs da metanálise de Borgia et al.,^3^ (mediana ou média de 113 a 192 minutos na maioria).^3^ O tempo da terapia lítica até a cinecoronariografia também foi maior no estudo brasileiro (mediana de 12 horas) do que nos ECRs da metanálise acima (tipicamente <5 horas).^3^ Por fim, o notável atraso de 71 (intervalo interquartil: 42-135) minutos desde a chegada na unidade de saúde até o início da fibrinólise na experiência brasileira é muito superior aos 20 minutos recomendados pelas diretrizes.^7^ Juntos, esses números fornecem subsídios para identificar alvos de melhoria na qualidade do atendimento de pacientes com IAMCSST no Brasil.

Um destaque do artigo publicado nos Arquivos Brasileiros de Cardiologia refere-se às diferenças sexuais no tratamento e prognóstico no cenário da estratégia farmacoinvasiva no IAMCSST. Em uma análise multivariada, o sexo feminino permaneceu como fator preditivo de mortalidade intra-hospitalar. Além disso, o estudo mostrou uma alta prevalência de sintomas atípicos e maiores demoras para procurar atendimento médico e iniciar fibrinólise após admissão no centro médico em mulheres,^6^ favorecendo um resultado adverso. Esses resultados estão alinhados com extensa evidência de pior prognóstico após infarto agudo do miocárdio e ICP em mulheres.^8–10^ Além disso, em pacientes internados por IAMCSST em hospitais com capacidade de ICP no estado brasileiro de Sergipe, foram observadas disparidades marcantes entre os sexos em relação à taxa de ICP primária (44% em mulheres e 54,5% em homens) e mortalidade intra-hospitalar (16,1% em mulheres e 6,7 % em homens).^11^ Considerando que a doença isquêmica do coração é a principal causa de morte no Brasil, compreendendo 12% dos eventos fatais em mulheres,^12,13^ esses achados justificam iniciativas como a Carta das Mulheres da Sociedade Brasileira de Cardiologia^14^ em um movimento para aumentar a conscientização de pacientes e médicos sobre a importância da doença cardiovascular em mulheres. Da mesma forma, a *American Heart Associatio n lançou recentemente o Call to Action for Cardiovascular Disease in Women*, com o objetivo de promover a equidade para as mulheres no contexto da saúde cardiovascular.^15^

Portanto, vários diagnósticos podem ser extraídos do abrangente artigo sobre a dinâmica da estratégia farmacoinvasiva no IAMCSST em um centro brasileiro. O desafio agora é transformar essa rica informação em melhores cuidados médicos.
