# Supplementary material for: Estratégia Farmacoinvasiva no Infarto do Miocárdio com Supradesnível do Segmento ST no Brasil: Sexo Feminino como Fator Prognóstico
Source: Arq Bras Cardiol. 2022 Nov 1;119(5):703–4. [Article in Portuguese] doi: 10.36660/abc.20220688 (PMC9750206; doi:10.36660/abc.20220688)
Supplement: Translation

# Translation: Pharmacoinvasive Strategy in ST-Elevation Myocardial Infarction in Brazil: Female Sex as a Prognostic Factor

**Authors:** Fernando Cesena

**Affiliations:** 1 Guarulhos SP Brazil Cenocor, Guarulhos, SP – Brazil

**Keywords:** ST Elevation Myocardial Infarction, Thrombolytic Therapy, Percutaneous Coronary Intervention, Sex Differences

Primary percutaneous coronary intervention (PCI) is the recommended therapy in patients with acute ST-elevation myocardial infarction (STEMI). In locations without immediate PCI capability or in the presence of an anticipated delay from hospital presentation to primary PCI >120 minutes, fibrinolysis is indicated. In these cases, invasive angiography and PCI 3 to 24 hours after fibrinolysis may improve the prognosis and are recommended as class 2a by the recent ACC/AHA/SCAI Guideline for Coronary Artery Revascularization.^1^ The rationale for early routine PCI after fibrinolysis, the so-called pharmacoinvasive strategy, is that lytic therapy provides adequate (TIMI 3 grade) blood flow in only 50-60% of the cases. PCI can then relieve residual stenosis and restore normal flow, which is related to the benefit of reperfusion in reducing mortality. Importantly, early invasive angiography should follow fibrinolysis independently of the resolution of the ST-segment elevation since electrocardiogram changes have poor accuracy in identifying adequate reperfusion.^2^

The recommendation for the pharmacoinvasive strategy is supported by several randomized controlled trials (RCTs) and meta-analyses demonstrating clinical benefits over previous standard therapy.^3^ Moreover, the STREAM trial provided evidence that pre-hospital fibrinolysis followed by PCI after 6 to 24 hours is as efficacious as primary PCI in STEMI patients who cannot undergo primary PCI within 1 hour after the diagnosis.^4,5^

The *Arquivos Brasileiros de Cardiologia* publishes an article thoroughly reporting the experience of a Brazilian university hospital with the pharmacoinvasive approach in STEMI.^6^ Although this observational work is completely different from previous RCTs concerning design, purpose, and study population, it is tempting to make some comparisons.

In-hospital mortality in the Brazilian study was 5.6%, which is higher than the 3.3% 30-day mortality reported in a metanalysis of RCTs^3^ but not so higher than the 4.6% 30-day mortality in the pharmacoinvasive arm of the STREAM trial.^4^ Notably, the time from the symptom onset to the fibrinolytic treatment was longer in the Brazilian study (median 222 minutes) than in the STREAM trial (median 100 minutes)^4^ or in the RCTs of the metanalysis by Borgia et al. (median or average from 113 to 192 minutes in most of them).^3^ The time from the lytic therapy to the coronary angiography was also higher in the Brazilian study (median 12 hours) than in the RCTs of the metanalysis above (typically <5 hours).^3^ Finally, the remarkable delay of 71 (interquartile range: 42-135) minutes from the arrival in the medical unit to the start of fibrinolysis in the Brazilian experience is much longer than the 20 minutes recommended by guidelines.^7^ Taken together, these numbers provide insights to identify targets for improvement in the quality of care of STEMI patients in Brazil.

A highlight of the article published in the *Arquivos Brasileiros de Cardiologia* refers to sex differences in treatment and prognosis in the setting of the pharmacoinvasive strategy in STEMI. In a multivariable analysis, female sex remained a predictive factor for in-hospital mortality. Also, the study showed a high prevalence of atypical symptoms and longer delays for seeking medical care and initiating fibrinolysis after admission to the medical center in women,^6^ favoring an adverse outcome. These results align with large evidence of worse prognosis after acute myocardial infarction and PCI in women.^8–10^ Moreover, in patients admitted for STEMI in hospitals with PCI capacity in the Brazilian state of Sergipe, striking sex disparities were observed regarding the rate of primary PCI (44% in women and 54.5% in men) and in-hospital mortality (16.1% in women and 6.7% in men).^11^ Considering that ischemic heart disease is the main cause of death in Brazil, comprising 12% of fatal events in women,^12,13^ these findings justify initiatives such as the Brazilian Society of Cardiology Women’s Letter^14^ in a movement to increase patient and physician awareness of the importance of cardiovascular disease in women. Similarly, the American Heart Association has recently issued the Call to Action for Cardiovascular Disease in Women, aiming to promote equity for women in the context of cardiovascular health.^15^

Therefore, several diagnoses can be retrieved from the comprehensive article on the dynamics of the pharmacoinvasive strategy in STEMI in a Brazilian center. The challenge now is to transform this rich information into better medical care.
